# Cognitive Functioning of Children in Out-of-Home Care

**DOI:** 10.1007/s40653-023-00580-8

**Published:** 2023-11-14

**Authors:** Misja Eiberg

**Affiliations:** VIVE - The Danish Center for Social Science Research, Herluf Trolles gade 11, 1052 , Copenhagen, Denmark

**Keywords:** Foster care, Maltreatment Effects, Cognitive Development, IQ, Executive Functioning, WISC IV, BRIEF, CNT

## Abstract

Purpose: Most children who enter out-of-home care (OHC) have been subjected to prolonged maltreatment. Maltreatment potentially contributes to a cumulative deficit in neurocognitive maturation and development that is likely to proceed with the child’s placement into OHC and persist throughout adulthood. From the theoretical perspective of how maltreatment may affect the developing brain, this study examines the IQ and executive function of children placed in OHC on standardized, norm-referenced measures. Furthermore, the study investigates the prevalence of serious cognitive delays, defined by scores in the clinical range on the administered instruments. Methods: The study included 153 children in foster care (66% female), aged 6–15 (*M* = 10.5, *SD* = 2.1). Independent two-sample t-tests were run to test for significant differences between the sample and the norm population on the applied neuropsychological measures. Results: The results showed that discrepancies in cognitive development were global in scope, with the children lagging significantly behind the norm population on all applied measures with discrepancies ranging from 0.61 to 2.10 *SD* (*p* < .001). Also, serious developmental delays in all cognitive domains were vastly overrepresented in the sample ranging from 11.3% (IQ) to 66.0% (executive function). Conclusions: The results document a very high prevalence of cognitive deficits and delays among the children in the sample. The implications of identifying the neurocognitive effects of maltreatment in the practices of the child welfare system are discussed in terms of developing suitable assessment and intervention strategies.

## Introduction

At the time of entry into out-of-home care (OHC), the vast majority of children have been subjected to prolonged maltreatment (Ankestyrelsen [The Danish Social Security Appeals Board], 2016; Burns et al., 2004; Drake et al., 2022; Oswald et al., 2010) often referred to as complex trauma (Cook et al., 2005; D’Andrea et al., 2012). Many children in OHC lack essential protective factors, such as secure attachment to a primary caretaker, making the child more vulnerable to the negative impact of maltreatment on development (Baer & Martinez, 2006; Breidenstine et al., 2011; Cook et al., 2005; Gerson & Rappaport, 2013; Mclaughlin & Lambert, 2017; Sattler & Font, 2018; Vasileva & Petermann, 2018). Chronic lack of adequate parental care with few opportunities for age-appropriate stimulation and/or a high risk of exposure to trauma poses a risk to neuropsychological development (Cassiers et al., 2018; De Bellis, 2005; Fox et al., 2011; Karatoreos & McEwen, 2013; Smith & Pollak, 2020; Teicher & Samson, 2016), which potentially contributes to a cumulative deficit in neural maturation that likely proceeds the child’s placement into OHC, and often persist throughout adulthood (De Bellis & Zisk, 2014; Dvir et al., 2014; Su et al., 2019; Widom, 2014). Childhood maltreatment has been associated with maladaptive neurodevelopment, such as accelerating neuron loss, delays in myelination, abnormalities in developmentally appropriate pruning, reduced cerebral and intracranial volume, changes in neuroendocrine and neurotransmitter systems, and inhibition of neurogenesis (Colich et al., 2020; De Bellis, 2005; Lim et al., 2014; McCrory et al., 2011; Nemeroff, 2016: Paquola et al., 2016; Peverill et al., 2023; Teicher et al., 2003; Teicher & Samson, 2016), in addition to deficits in cognitive functioning including IQ, executive functioning (EF), language development, learning, and memory (Davis et al., 2015; Fry et al., 2016; Kavanaugh et al., 2017; Kirke-Smith et al., 2012; Lum et al., 2015; Masson et al., 2015; Sylvestre et al., 2016; Watts-English et al., 2006; Young-Southward et al., 2020). Neglect is the most prevalent type of maltreatment among children who enter the care system and often co-occurs with other types of maltreatment and traumas (Ankestyrelsen [The Danish Social Security Appeals Board], 2016; Bullinger et al., 2020; McLaughlin et al., 2014; U.S. Department of Health and Human Services, 2021; UK Department of Education, 2019). For instance, a US national study measuring seven adverse childhood experiences (ACEs), such as parental abuse, violence exposure, or household member mental illness or substance abuse, showed that for children with care experience, the average number of ACEs prior to entering OHC was 2.5 compared to 0.6 for children who were never in OHC (Turney & Wildeman, 2017).

Despite the growing awareness and mounting evidence of the neurocognitive ramifications of childhood maltreatment throughout development, less is known about differences in neurocognitive outcomes of children in OHC and how these differences may affect the variance in long-term outcomes. In addition, differences in the care experience may also curb or add to the adverse experience of maltreated children, potentially diverting or reinforcing a poor developmental trajectory (Leloux-Opmeer et al., 2016; Oosterman et al., 2007; Van Holen et al., 2019). Generally, research documenting the often poor life outcomes for OHC alums, for example, in education and occupational attainment (e.g., Gypen et al., 2017), has grown much more rapidly than research investigating the reasons for this long-term divide. However, differences in neuropsychological development, for instance, in IQ, are an important long-term predictor of many psychosocial outcomes such as educational achievement (e.g., Brännström et al., 2020).

Evidence about the cognitive functioning of children who are placed in OHC can provide insight into the diverse effects of childhood trauma and maltreatment on the developmental trajectory even after the ceasing of the maltreatment and placement into OHC, and, thus, contribute to a better understanding of the mechanisms behind the vast achievement and life quality gap between OHC alums and the general population over the life course (Brännström et al., 2017; Gypen et al., 2017; Kääriälä & Hiilamo, 2017; Zlotnick et al., 2012).

This study investigates the cognitive functioning (IQ and EF) of 153 children in OHC and discusses the results from the point of environmental interaction in brain development. The findings contribute to the mounting international evidence of the developmental disadvantage of children in the care system and focus on clinically important cognitive differences in the sample by investigating the prevalence of serious cognitive delays and scores of clinical concern indicative of functional impairment. Furthermore, and perhaps even more importantly, these results could guide the development of interventions and practices in the care system to support developmental growth in maltreated children while in OHC.

### Sensitive Periods and Environmental Interaction in Brain Development

Early development is characterized by sensitive periods in which certain environmental experiences are particularly important to nurture the maturation of the brain, and others are particularly harmful to neural development (Andersen, 2003; Greenough & Black, 1992; Knudsen, 2004; Nelson & Gabard-Durnam, 2020). Sensitive periods are concentrated in early childhood, where most experiences are mediated to the child by the primary caregiver(s), emphasizing the importance of that relationship on the neural organization (Glaser, 2000; Schore, 2001). How needs, challenges, and developmental tasks are met and accomplished during different developmental stages determine the neural integration of experiences and later adaptation (Cicchetti & Tucker, 1994; Fox et al., 2010; Glaser, 2000; Greenough et al., 1999; Munakata et al., 2004; Tau & Peterson, 2010). Greenough and colleagues (Greenough & Black, 1992; Greenough et al., 1987) describe the dynamic of environment interaction and neural integration through two main developmental processes, both of which rely on sensitive periods. E*xperience-expectant* development is defined as stages in neural development from birth to early adulthood that will not happen unless a certain species-typical experience (e.g., particular sensory stimulus) occurs at a particular time during development (a sensitive period), and this early acquisition is essential for further development. If expectant experiences do not happen, the development will depend on whatever aberrant information experience does provide, and hence may lead to maladaptation in the neural organization such as excessive pruning of neurons. *Experience-dependent* development is defined as neurological non-predetermined development that depends on individual interactions with the environment to modify and form new synapses throughout life. How individual environmental interaction influences the plasticity of the developing brain interplay with sensitive periods in neural maturation (ibid.). The developmental model of Greenough and colleagues emphasizes the significance of environmental deprivation and adverse experience on neural organization and demonstrates how prolonged interaction with abusive and/or neglectful caregivers affects neurodevelopment and ultimately alters the physiology of the brain (Bick & Nelson, 2016).

From the perspective of environmental interaction, maltreatment by neglect may repress stages in neurodevelopment reliant on expected experiences; for example, the development of motor skills or maturation of sensory systems contingent on adequate sensory stimulation from the environment (e.g., physical touch or visual stimuli). It has also been suggested that early language acquisition (phonetic awareness) and attachment could be aspects of early social development reliant on the expected common experience of a suitable caregiver, which is often not available to deprived and neglected children, though the mechanisms are not well understood (Joseph, 1999; Twardosz & Lutzker, 2010). Lack of interactions and inadequate emotional engagement by a neglectful caregiver may also result in scarcity of experience-dependent development, for example, by lack of novel opportunities for learning and social interaction, and hence repress neural growth.

Furthermore, a child-caregiver relationship characterized by abuse, such as violence, also induces environmental interactions harmful to experience-dependent development, for example, by inappropriate external modulation of the child’s affect by the aggression of the caregiver, which can be especially harmful at a particular time in development were sensitive affect regulation by the primary caregiver(s) is vital to form self-regulatory capacities and attention control (Berger, 2011; Bernier et al., 2010; Kalbfleisch, 2017). Moreover, differences in environmental interaction related to different types of maltreatment (e.g., neglect vs. physical abuse) may also explain the characteristic developmental differences, which have often been found in maltreated children depending on the type of maltreatment they have been subjected to and the timing of the maltreatment (Cowell et al., 2015; Jaffee & Maikovich-Fong, 2011; Kavanaugh et al., 2017; Keiley et al., 2001; Machlin et al., 2023; Manly et al., 2001; McLaughlin et al., 2014). For instance, children who were neglected tend to have poorer cognitive function than children who were abused, but not neglected (see e.g., Geoffroy et al., 2016), and generally maltreatment during multiple developmental periods and multiple types of maltreatment or traumas predict worse developmental outcomes (Brown et al., 2019; Hughes et al., 2017; Jaffee & Maikovich-Fong, 2011; Manly et al., 2001; Vivrette et al., 2018). The multidimensional impact on neural organization through environmental interaction exposes the global range of potential effects of maltreatment on neuropsychological development and explains why some of these effects are likely to persevere after the ceasing of the maltreatment and the child’s placement into OHC.

### The Current Study

This present study examines the neuropsychological outcomes of fostered children on standardized and norm-referenced measures of IQ and EF and compares these to the outcomes of the norm samples of the measures. To study the scope of serious developmental delays among the children in OHC, the prevalence of extreme outcomes was examined based on clinical cutoff scores of the applied instruments.

The main hypothesis is that (a) the children in OHC would perform significantly below the population norm on all the included measurements consistent with the empirical and theoretical literature on maltreatment, and (b) that serious developmental delays also would be more common among the children in foster care than in the norm population.

## Materials and Methods

### Participants

The study included 153 children (66% female), aged 6–15 (*M* = 10.5, *SD* = 2.1), and living in foster care across 24 Danish municipalities (a quarter of all Danish municipalities), including both urban and rural areas. The data was collected in 2014 as pre-treatment data in a randomized controlled trial (RCT) of scholastic support interventions for children in OHC attending regular schooling (Eiberg & Scavenius, 2023a, 2023b). The RCT’s inclusion criteria included placement in foster care and attendance in grades 1–7 in a regular school. Exclusion criteria included pre-trial diagnosed pervasive developmental disorders (defined by code F84 in ICD-10 in 2014) or intellectual disability (defined as IQ < 70) and pre-trial planned reunification that was to take place during the duration of the RCT. The foster caregivers provided the information. Though pre-trial diagnosed intellectual disability was an exclusion criterion in the initial RCT it is still considered relevant to investigate the prevalence of serious cognitive delays, since children in the child welfare system may be underdiagnosed.

The children had been in their current placement for an average of 5.5 years (*SD* = 3.5). According to the foster caregivers, 8.5% of the children (8.2% of the girls and 9.1% of the boys) had one or more psychiatric diagnoses, including ADHD, reactive attachment disorder, Tourette’s syndrome, anxiety, and fetal alcohol spectrum disorders.

### Measures

Data was collected in 2014 using the most recent version of selected instruments available at the time in validated Danish translations. The psychometric properties of the instruments are available in the listed references. Where possible, published Danish norm populations were applied for comparison. The data was collected by trained psychologists.

**The Wechsler Intelligence Scale for Children IV (WISC IV).** The Wechsler Intelligence Scale for Children IV (WISC IV) was used to measure intellectual ability (IQ). WISC IV is a comprehensive, standardized, and norm-referenced measure to estimate IQ. The full-scale IQ score (FSIQ) comprises 15 subtasks in four distinct cognitive domains, including the Verbal Comprehension Index (VCI), the Perceptual Reasoning Index (PRI), the Working Memory Index (WMI), and the Processing Speed Index (PSI). It uses standardized scores with a mean of 100 and an *SD* of 15. The normal range of IQ is defined as mean ± 1 *SD* (i.e., scores ranging from 85 to 115) (Wechsler, 2003). The Danish norm population consists of 477 individuals (52.4% boys) aged 6–16 years.

**The Behavioral Rating Inventory of Executive Function (BRIEF).** The Behavioral Rating Inventory of Executive Function (BRIEF) is a standardized, norm-referenced questionnaire with 86 items covering a range of behaviors related to executive functioning (e.g., self-monitoring and impulse control) rated on a three-point scale (never, sometimes, and often). The BRIEF generates two index scores, namely a Metacognition Index (MI) and a Behavioral Regulation Index (BRI), as well as a full-scale score, the General Executive Composite (GEC). The BRIEF uses T-scores with a mean of 50 and an *SD* of 10 and has a clinical cut-off t-score of 65 (1.5 *SD* above average). Percentiles for all scale scores are available (Gioia et al., 2000). The BRIEF was administrated to foster mothers, foster fathers, and teachers who responded concerning the children. Though BRIEF was available in Danish, there was no Danish norm population for the BRIEF. For the analysis, US norms were applied, which include 1.419 individuals (43% boys) aged 5–18 years for parent rating, and norms for teacher ratings included 720 individuals (44% boys).

**The Contingency Naming Test (CNT).** The Contingency Naming Test (CNT) is a standardized, norm-referenced performance-based test of EF that resembles the classic Stroop test but entails two shifting conditions and comprises colored geometric figures instead of colored words (Anderson et al., 2000, 2001). The CNT requires visual attention, cognitive flexibility, inhibitory control, and working memory, and its primary outcome is an efficiency score, which is a combined measure of time use and accuracy, with higher scores indicating better functioning. Children aged six (*n* = 2) are excluded from the analysis since there are no norm data for this age group. There are currently no Danish norm data available for the CNT, and the analysis applies Australian norm data, including 381 individuals (49.6% boys) aged 7–15.

## Procedure

### Statistical Analysis

Descriptive statistics were obtained on all measures to explore the developmental status of the children. T-tests of independent samples using means and SDs were run to test for significant differences between the sample means and norm population means on the applied measures. The significance level was set at 5%. For the CNT z-scores were formed, standardizing the difference between each child’s score and the population mean with a standardized mean of 0 and an *SD* of 1.

The prevalence of serious developmental delays was defined by clinical cutoff scores available for each test:

- Cognitive delay: FSIQ score < 70 in WISC IV.
- Executive functioning of clinical concern: GEC T-scores ≥ 65 on the BRIEF. In addition, the percentage of children scoring at the very top of the distribution ≥ 97th percentile in the BRIEF is also reported.

The CNT norms do not have a cut-off score or percentiles to indicate extreme scores and are, therefore, not included in this analysis.

## Results

### Developmental Outcomes of Children in OHC

Table 1 shows a summary of key descriptive statistics and t-test results for all included outcomes for the full sample.

**Table 1:** Means, Standard Deviations, Min. and Max Scores, and group Differences for Cognitive Outcomes for Children in Out-of-Home Care and the Norm Population

	*n*	*M*	*SD*	Min.	Max	*MD*	95% CI for *MD*	*t*
**Intellectual ability**								
*WISC IV: Full-scale IQ (Stand. score)*	151	87.61	13.85	42	115	-12.39	[-9.69; -15.09]	9.01***
**Executive functioning**								
***BRIEF***: *foster mothers (T-Score)*:								
*Global Executive Composite (GEC)*	150	67.61	9.08	49	87	17.61	[15.94; 19.28]	20.68***
*Behavior Regulation Index (BRI)*	150	66.49	11.19	40	91	16.49	[14.79; 18.19]	18.98***
*Metacognition Index (MI)*	150	66.47	8.54	45	86	16.47	[14.81; 18.13]	19.44***
***BRIEF***: *foster fathers (T-Score)*:								
*Global Executive Composite (GEC)*	124	66.31	9.50	40	91	16.31	[14.48; 18.14]	17.49***
*Behavior Regulation Index (BRI)*	124	64.78	11.41	41	91	14.78	[12.92; 16.64]	15.60***
*Metacognition Index (MI)*	124	65.58	9.08	39	86	15.58	[13.76; 17.40]	16.76***
***BRIEF***: *teachers (T-Score)*:								
*Global Executive Composite (GEC)*	150	70.37	17.14	42	117	20.37	[18.34; 22.40]	19.66***
*Behavior Regulation Index (BRI)*	150	70.95	19.95	43	122	20.95	[18.82; 23.09]	19.23***
*Metacognition Index (MI)*	150	67.72	15.49	42	109	17.72	[15.76; 19.68]	17.73***
***CNT***: *Efficiency score (z-score)*	119	− .61	0.91	-2.20	3.93	− .61	[-.81; − .41]	5.93***

*** p < .001

Note. WISC IV = Wechsler’s Intelligence Scale for Children IV. BRIEF = Behavioral Rating Inventory of Executive Function. CNT = Contingency Naming Test. M = Mean. SD = Standard Deviation. MD = Mean Difference. CI = Confidence Interval

**Intellectual ability (IQ).** The result of the t-test shows a significant discrepancy between the FSIQ scores of children in the sample and the norm population (Table 1). The sample average FSIQ score of 87.61 (*SD* = 13.85, min. = 42, max. = 115) falls 0.83 *SD* under the average and is within the range of normal IQ (85–115), but in the lower end of the range. Moreover, 36.4% of the children obtained an FSIQ score below the normal range of intellectual ability (< 85) (not shown).

**Executive functioning**. Overall, the prevalence of deficits in executive functioning was very high in the sample, especially when EF was measured by the BRIEF. The results of the t-tests (Table 1) show that average scores on the BRIEF, i.e. the GEC, and the two subscales MI and BRI all are significantly above the population mean for ratings by foster mothers, foster fathers, and teachers. Further, all average scores are above the clinical cut-off score of 65 (1.5 *SD* above average), except the BRI when rated by foster fathers, where the average score is just below the clinical threshold (*M* = 64.78, *SD* = 11.41). Overall, average scores range from 1.48 to 2.10 *SD* above the norm average.

The results also showed that the average efficiency score obtained by the CNT is significantly below the norm population mean (Table 1). CNT holds no cut-off score, but the average z-score of -0.61 (*SD* = 0.91) is noticeable below the standardized norm population mean of 0, indicating that the children are demonstrating a clear disadvantage in complex tasks involving cognitive flexibility, visual attention, working memory, and control of involuntary responses compared to other children of their gender and age.

### Serious Developmental Delays

**Cognitive delay (IQ).** Overall, 11.3% of the children in the sample obtained an FSIQ score < 70, indicative of intellectual disability (Disorder of Intellectual Development, ICD 11) even though pre-diagnosed intellectual disability was an exclusion criterion in the RCT study the children were sampled for.

**Executive functioning.** The overall percentage of children obtaining a score on the GEC in the BRIEF above the clinical cut-off (≥ 65 or 1.5 *SD* above the mean) was 66% for ratings by foster mothers, 61.3% for ratings by foster fathers, and 59.3% for teacher evaluations. Regarding the children obtaining scores at the top of the GEC distribution in the BRIEF, 40.0% of the children obtained a score ≥ 97th percentile based on teacher ratings, and 25.0% and 30.7% based on ratings by foster mothers and foster fathers, respectively.

## Discussion and Conclusions

The results of the analysis and their interventional implications are discussed for each outcome, and the section concludes with a discussion of future directions to address the practical implications of the findings for the care system.

### Intellectual Development

Based on FSIQ scores, children with an intellectual disability in this sample comprised 11.3% compared to approximately 1% of the general global population of children and youth (Maulik et al., 2011). Moreover, the results suggest a discrepancy in the average FSIQ score between the children in the sample and the general population of 0.83 *SD*. Very similar discrepancies in the intellectual development of maltreated children and youth in the child welfare system have been consistently demonstrated in previous research conducted over the last decades. For instance, Evans (2001), De Bellis et al. (2009), Kirke-Smith et al. (2014), Pears et al. (2010), Tordön et al. (2020), Viezel et al. (2015), Vinnerljung and Hjern (2011) applied a measure of general cognitive ability on samples of maltreated children and youth mostly residing in foster care, and all found discrepancies in cognitive abilities ranging from 0.6 *SD* to 0.8 *SD*. The consistency of these results across studies may stem from the generally high prevalence of neglect in OHC samples (see e.g., Oswald et al., 2010). Neglect causes deprivation of environmental experience necessary to enhance and mature cognition such as social interaction and sensory stimulation, and thus neglect is more often associated with poorer cognitive outcomes than other types of maltreatment, for example, physical abuse (Cicchetti & Valentino, 2015; Font & Berger, 2015; McLaughlin et al., 2017). Though some examples of similar studies find smaller discrepancies in cognitive development (e.g., Perzow et al., 2013), the rather consistent findings of cognitive deficits among children in OHC propose an important compensatory aim of the care system. This is particularly relevant considering the disadvantage of poor cognitive functioning on many long-term outcomes such as incarceration (e.g., Beaver et al., 2013) and educational attainment (e.g., Brännström et al., 2020; Hegelund et al., 2018).

**Interventional implications.** To enhance cognitive outcomes, some structured cognitive training programs for children hold promise, and several studies have shown positive results on functions such as fluid reasoning (e.g., Nutley et al., 2011) and working memory (e.g., Holmes et al., 2009). However, research in this field is still scarce, and it is largely unknown whether the benefits of training apply to maltreated populations and whether the cognitive gains from specific training programs increase general learning and adaptation over time. In addition to content-specific training programs, evidence from adoption and foster care studies suggests that repressed intellectual ability can potentially recover or improve through environmental interaction in later childhood (Almas et al., 2016; Christoffersen, 2012; Fox et al., 2011; Nelson et al., 2007; Van Ijzendoorn et al., 2005; van Ijzendoorn & Juffer, 2006). Studies of adoptees show that adopted children, on average, demonstrate a relative cognitive catch-up over time compared to biological siblings who remained in their birth home or stayed institutionalized (Christoffersen, 2012; Van Ijzendoorn et al., 2005; van Ijzendoorn & Juffer, 2006). Moreover, children in residential care show rapid brain growth and increased cognitive development when deinstitutionalized in foster care, the birth family or an adoptive family (Van Ijzendoorn et al., 2020). Even grossly neglected and deprived institutionalized children have shown significant changes in brain development and cognitive improvement over time when placed in long-term foster care compared to peers who remained institutionalized (Almas et al., 2016; Fox et al., 2011; Humphreys et al., 2022; Sheridan et al., 2012). In addition to a potential naturalistic effect of long-term or permanent placement, there is relatively unexplored potential in working systematically with foster families and residential care staffers to increase the overall compensatory effect of the placement on cognitive functioning through environmental enrichment. Accelerating experience-dependent development for children in both long- and shorter-term OHC through targeted interventions, especially for children with serious cognitive delays, may improve long-term developmental outcomes. Still, such interventions appear to be a somewhat unexplored field. For instance, a recent meta-analysis of 53 studies on parent-child interventions for adoptees and children in foster care includes no cognitive outcomes (Schoemaker et al., 2020). Comprehensive interventions developed for OHC settings typically focus primarily on emotional and behavioral trauma recovery and do not target general development (e.g., Trauma-Focused Cognitive Behavioral Therapy or Treatment Foster Care) (Fisher et al., 2009; Fratto, 2016; Leve et al., 2012; Racusin et al., 2005).

### Executive Functioning

The results confirm the initial hypothesis that the children in the sample have significantly poorer EF than the norm population on both behavioral and performance-based measures of EF, though the discrepancy in average scores on the behavioral ratings on the BRIEF is noticeably larger (discrepancies ranging from 1.48 to 2.10 *SD*) than the discrepancy on the performance-based measure, CNT (discrepancy = 0.61 *SD*). However, of the 142 children who were tested with the CNT, only 119 completed all four subtests and obtained a total score. For comparison, the mean z-score of the third trial of the CNT (one shifting condition) was − 0.72 (n = 142), and the mean z-score of the fourth trial (two shifting conditions) was only − 0.46 (n = 119) despite it being the most complex task in the CNT. It is, therefore, likely that the poorest performers in the CNT were not included in the analysis of the total score, resulting in a better average score across the four subtests of the CNT. Still, differences in behavioral ratings and performance-based measures of EF have also been found in other studies of both clinical and non-clinical samples (Fay-Stammbach & Hawes, 2019; Toplak et al., 2013), presumably because rating scales capture more aspects of daily function, including complex behavior such as goal-directed behavior, which is not captured by performance measures (Suchy et al., 2017; Toplak et al., 2013). A review by Toplak and colleagues (2013) of the constructs and correlations of behavioral and performance-based measures of EF generally finds a weak correlation between BRIEF and performance measures, one of the probable reasons being that several of the scales included in BRIEF (such as the “initiate” scale) have no parallel performance-based measure. As Toplak et al. (2013) concluded, it is reasonable to assume that the different modalities of the EF measures, like those applied in this study, capture different aspects of functioning under different conditions.

The children in this study demonstrate a clear disadvantage on both behavioral and performance measures of EF. It is also noticeable that, regardless of the respondent, the highly elevated average scores on the BRIEF across all domains suggest severe problems with self-regulation and metacognition both at home and in school. The slightly higher average scores from teacher ratings may reflect the structured context of school being more demanding on EF than the foster care environment at home. Overall, the prevalence of children obtaining GEC scores on the BRIEF of clinical relevance is extremely high, and the children in the sample were also vastly overrepresented at the very top of the GEC distribution, despite only one child in the sample having an ADHD diagnosis. Generally, studies in the field of OHC have also found a clear discrepancy in EF between children in OHC and the general population on diverse measures (Fry et al., 2016; Lansdown et al., 2007; Lind et al., 2017; Pears & Fisher, 2005), as have developmental studies of maltreated children and youth (Carvalho et al., 2020; Kavanaugh et al., 2017; Kirke-Smith et al., 2012, 2014; Malarbi et al., 2017). As with general cognitive delay, executive dysfunctioning is especially associated with neglect (Johnson et al., 2021) but is generally predicted by multiple forms of maltreatment and trauma exposure (Lund et al., 2020), which may explain the vast overrepresentation of children in OHC with executive dysfunction.

**Interventional implications.** The accumulating evidence of generally poorer executive functioning among children who enter the care system should compel OHC professionals and foster caregivers to raise awareness of potential impairment in EF when intervening in school or addressing behavioral problems. Although EF impairments and behavioral problems (e.g., impulsive and disruptive behavior) often appear simultaneously in children, failing to draw a clear conceptual distinction might lead to misperceptions of EF impairments as a problem behavior and not a deficit in cognitive functioning. When EF impairment is addressed in the current literature on children in the child welfare system, there is a tendency to consider EF impairment and certain behavior problems collectively (see, e.g., O’Higgins et al., 2017). Though inexpedient, this practice actually mirrors the dynamics of many child-focused EF intervention models that typically aim at EF through training of certain behaviors and skills (Bierman & Torres, 2015; Cortese et al., 2015; Diamond & Lee, 2011), and where the progress often relies primarily on the child’s efforts to change behavior, in some cases working completely independently (e.g., computerized training). Research into primarily behavior-based and individualized interventions (such as computer-based training, mindfulness practices, aerobics, and attentional control training) for children with or without ADHD shows mixed results on self-regulatory processes and working memory. However, the mechanisms are poorly understood, and long-term effects on behavioral adaptation and learning are understudied and generally less promising (ibid.).

Viewing the development of executive functioning on the basis of environmental interaction in brain development (Greenough & Black, 1992) and evidence of neurohormonal dysregulation due to chronic activation of the stress-response system (e.g., De Bellis & Zisk, 2014), much of the regulatory processes that are malfunctioning in maltreated children with EF impairments relate to inadequate and abnormal environmental interaction in early childhood, including chronic lack or inappropriate external regulation of arousal. This interpersonal developmental dynamic suggests that treatment based on *relationships* that aim to nurture emotional processing and the regulation of arousal and attention—and not regulate a particular expressed behavior—could potentially be more effective than individualized training to treat EF impairment. Though there is not yet much evidence for the different treatment programs aimed at EF, it does seem that relational programs in naturalistic settings hold more promise for generalized gains (Bierman & Torres, 2015). From that perspective, a few current trauma-informed treatment models suitable for foster care settings have shown promising results in reducing EF impairment, such as the “Attachment and Biobehavioral Catch-up for Toddlers” (ABC-T) (Lind et al., 2017). The ABC-T intervention bases building self-regulatory capacities on interpersonal experiences and strengthened security of the relationship between the child and the foster caregiver (Dozier & Bernard, 2017).

### Conclusions and Implications for OHC Practice

Since this sample originated from an RCT of school support interventions for children in OHC, children struggling in school may have been overrepresented. However, the present results broadly confirm the current evidence in the research literature concerning the neuropsychological ramifications of childhood maltreatment in general, with maltreated children, on average, scoring below the population mean on cognitive measures. Yet, what is particularly important about the present findings is the very high prevalence of children not only scoring below the mean but obtaining scores indicative of functional impairment.

The results suggest that the children’s development and learning, in many cases, require additional targeted support in the foster home and in school from the time of entry into OHC to prevent accumulating problems with educational attainment or functioning over time.

The model of environmental interaction in brain development implies that not only the type and timing of the maltreatment prior to the placement but also the quality and timing of the placement itself matter to the neurobiological developmental trajectory of children who enter the care system. Hence, later timing of the placement puts the developing brain at greater risk due to the accumulating negative effect of maltreatment on development over time. Accordingly, children who were placed in OHC in later childhood and adolescence often show poorer outcomes than children who were placed in earlier childhood (Dregan & Gulliford, 2012). However, some studies have also shown that children who were placed in OHC had outcomes somewhat poorer than or similar to children at risk who stayed at home, especially when placement occurred late (Doyle, 2007; Goemans et al., 2016). Besides the potential effect of the placement timing disadvantaging older children, the general poor or ambiguous outcomes of OHC may stem from the sometimes inadequate developmental conditions of OHC, especially concerning the inconsistent quality of the child-caregiver relationship. For instance, many children in OHC experience placement instability, that is, in the form of multiple placements and reunifications (Bell & Romano, 2017; Goering & Shaw, 2017; Jedwab & Shaw, 2017), placement breakdown (Oosterman et al., 2007; Vanderfaeillie et al., 2018), and/or periods in residential or group home care (Leloux-Opmeer et al., 2016) where the caregiver-child relationship is disrupted or lost, characterized by conflict, or subject to the restrictions of the residential/group home care setting. Moreover, previous placement breakdown increases the risk of new breakdowns constituting a potential further cumulative risk to development (Rock et al., 2013). Since the child-caregiver relationship is essential for the environmental interaction in brain development, the relational conditions for some children in OHC seem to have limited potential for providing an apt environment to accelerate developmental growth despite the placement protecting the child from further abuse. In fact, the lack of a consistent, suitable caregiver in the care system over time most likely adds to the developmental experience of adversity (Leloux-Opmeer et al., 2016; Mcguire et al., 2018; Okpych & Courtney, 2017; Wade et al., 2019). This seems to be especially true for older children with late entry or multiple entries, who are more likely than young children to experience placement instability or breakdown and/or residential/group home care, which perhaps comes on top of the increased developmental risk with higher age at the time of the placement. Plausibly, the general earlier timing of the placement and a consistent caregiver are the main reasons why developmental outcomes over time are often found to be better for adoptees compared to children in the care system (e.g., Vinnerljung & Hjern, 2011). However, there is no evidence to suggest that scarce progression in a child’s developmental outcomes in OHC indicates that the child would have been better off at home or that well-thought-out changes in the placement cannot be for the better. On the contrary, variating relational quality is a well-documented fundamental challenge for the care system that needs addressing to improve the developmental outcomes of all children in OHC, especially children who enter late and who may have developed more maladapted behaviors (James, 2004; Rock et al., 2013). Providing stable, consistent, and secure relationships during the placement, regardless of whether the placement is long- or short-term, is essential to improving the developmental outcomes of OHC in addition to preventing harm.

Still, in this study, the children’s average placement time in their current placement was more than five years, suggesting that the developmental problems persist even after many years in a stable placement. This is important to consider because maltreatment, despite the best efforts of the care system, often has a lasting impact on children’s development and long-term outcomes. Nevertheless, in the previous paragraphs, I argue that there is an insistent need for novel intervention strategies during the placement to improve the compensatory effect of OHC on cognitive development centered on strengthening and qualifying the child-caregiver relationship (such as the abovementioned ABC-T program) in order to support development and prevent placement breakdown. Moreover, for such interventions to be successful, the care system must commonly recognize the comprehensive ramifications of childhood maltreatment in its practices, and systematic assessment of development and functioning is key to improving treatment and training. Systematic assessment of children entering OHC can promote a shift from the narrow interventional focus on trauma-related or behavioral symptoms towards a broader focus that includes the child’s general developmental needs and the comprehensive care environment (Zilberstein, 2021). Perry and colleagues have done profound work in this field, developing the “Neurosequential Model of Therapeutics,” which applies neuroscience principles to structure clinical assessment and identify suitable intervention strategies for traumatized and maltreated children (e.g., Perry, 2009).

Unfortunately, many countries currently have no formal process for systematically assessing the developmental needs (emotional, cognitive, or otherwise) when a child enters OHC. In that regard, it is noteworthy that the IQ of the children in the present study with an IQ score < 70 was not previously documented, suggesting poor due diligence of assessment and lack of attention to developmental cognitive problems in the child welfare system.

Moreover, despite essential efforts to render a classification of the complex symptomology following chronic maltreatment in the ICD-11, the diagnosis of Complex Post-Traumatic Stress Disorder (C-PTSD) falls short of the neurocognitive developmental effects following prolonged or repeated traumatic stress. C-PTSD comprises criteria for affective and interpersonal dysfunction but does not include neurocognitive ramifications, particularly dysfunctions unrelated to the specific trauma symptomatology (*WHO|International Classification of Diseases, 11th Revision (ICD-11)*, 2019). C-PTSD provides a new important classification of some central maltreatment effects, but typical neurocognitive dysfunctions could be passed over in assessment and hence overlooked when determining treatment and care needs. As pointed out by Spann and colleagues (2012), maltreated children may not meet the criteria for any diagnosis but they might still have specific cognitive dysfunctions associated with the ramifications of the maltreatment they endured that persist at least into adolescence.

Concluding, future efforts in research and practice should focus on developing and evaluating OHC interventions to strengthen the child-foster caregiver relationship and to enrich the care environment to promote learning and cognitive development. In addition, to accommodate individual developmental needs and to provide better support and training for foster caregivers taking care of children with potential cognitive delays or impairment, developmental assessment of children entering care should be pursued as the standard procedure for every child.

### Limitations

A significant limitation of the study design is the self-selection of participants, who were recruited to participate in a randomized trial of school support interventions. Also, the design lacks a sampled non-OHC comparison group rather than relying on norm data for comparison. In addition. Danish norms were not available for the BRIEF and CNT. Furthermore, there is cumulating evidence that outcomes of childhood maltreatment vary with many factors, including the type of maltreatment, onset, severity, and duration, which have been found to cause different patterns of neuroplasticity as well as to moderate the neurological effect of maltreatment in different ways (De Bellis & Kuchibhatla, 2006; Gerson & Rappaport, 2013; Kavanaugh et al., 2017; Kirke-Smith et al., 2012; Manly et al., 2001; Teicher & Samson, 2016). Unfortunately, our records were not detailed enough to code variables relating to the maltreatment and placement history of the children to investigate potential differences in outcomes.
